# Clinical Validation of Quality Indicators for the Pharmacological Management of Chronic Non-Cancer Pain in Older Adult Inpatients

**DOI:** 10.2147/JPR.S560808

**Published:** 2026-02-02

**Authors:** Jasmin Abderhalden, Carla Meyer-Massetti, Danja Müller, Patricia Cadisch, Dominic Bertschi, Aljoscha Noël Goetschi

**Affiliations:** 1 Clinical Pharmacology and Toxicology, Department of General Internal Medicine, Bern University Hospital, Bern, CH-3010, Switzerland; 2 Institute of Primary Health Care (BIHAM), University of Bern, Bern, CH-3012, Switzerland; 3 Institute for Hospital Pharmacy, Bern University Hospital, Bern, CH-3010, Switzerland; 4 Department of Geriatrics, Bern University Hospital, Bern, CH-3010, Switzerland; 5 Graduate School for Health Sciences, University of Bern, Bern, CH-3012, Switzerland

**Keywords:** medication safety, chronic non-cancer pain, older adults, clinical pharmacy, quality indicators

## Abstract

**Purpose:**

Chronic non-cancer pain (CNCP) affects up to 88% of older adults. Pharmacological therapies are frequently prescribed long-term despite being considered second-line treatments and carrying significant risks, particularly for older adults. The present study aimed to clinically validate a set of quality indicators (QIs) that are used to improve the detection of medication-related problems during medication reviews performed by pharmacists for older adult inpatients with CNCP.

**Patients and Methods:**

We evaluated a set of medication review QIs—with previously established face and content validity—for a population of older adult inpatients diagnosed with CNCP. Subjects were in geriatrics wards in a tertiary hospital in Switzerland. Over two phases, pre- and post-QI implementation (Phase 1: July 2023 to April 2024, Phase 2: January to May 2025), pharmacists evaluated QIs for their applicability to the population, potential to improve medication reviews, acceptability by pharmacists, implementation issues and impact. These domains were used to evaluate the measurement properties of QIs in clinical practice, acting as proxies for clinical validity. Evaluations were interpreted using descriptive statistics.

**Results:**

We included 89 patients in phase 1 and 48 in phase 2. Of the 38 QIs evaluated, 27 (71%) were applicable to ≥ 5% of patients, 21 (55%) showed the potential to improve medication reviews, 12 (32%) revealed relevant treatment discrepancies that pharmacists accepted, 6 (16%) were rated as problematic to implement, 10 (37%) were impactful according to our predefined criterion and 6 (16%) met all criteria.

**Conclusion:**

The clinical evaluation of our set of previously developed QIs could help pharmacists to efficiently detect medication-related problems among vulnerable older adult populations with CNCP. The fact that only a few of the QIs were clinically valid means that future research is required, including the measurement of the influence of these QIs on patient-reported outcomes.

## Introduction

Chronic non-cancer pain (CNCP), which is defined as pain that lasts or recurs for more than three months and is not caused by a malignancy,^1^ affects 28–88% of older adults, making it a major public health concern.^2^ CNCP is complex. Indeed, 88% of patients with CNCP have multiple other chronic diseases.^3^,^4^ Depression, insomnia and other cognitive and physical disorders are common.^5–8^

Multimodal therapeutic approaches are generally recommended to account for the complexities of CNCP. Non-pharmacological interventions, including both psychological and physical therapies, make up first-line treatment choices. Pharmacological therapies should be considered second-line treatments.^9^ However, contrary to these recommendations, many older adults are treated pharmacologically, putting them at a higher risk of medication-related problems (MRPs).^8^ Older adults with CNCP often suffer from other chronic diseases,^3^,^4^ accordingly, polypharmacy is common,^8^ which further increases the risk for MRPs in these patients.^10^ This is particularly important because older adults with CNCP are often frail and therefore more susceptible to medication-related harm.^11^ Thus, quality improvement strategies are needed for this vulnerable patient group.

Quality indicators (QIs) are measurement tools that help check and compare the quality of care within and between healthcare facilities.^12^ They support the standardisation of care and help improve its delivery.^12^ When used with (electronic) decision-making tools, QIs’ triggers can help to quickly identify patients who may be at risk of poor care.^13^ Alternatives to QIs in electronic algorithms include targeted chart reviews, which require more personnel and time, and analysis of spontaneous reporting systems, which lack a systematic approach and tend to underreport MRPs.^14^,^15^ QIs are thus key to continuously improving healthcare, and they must be developed carefully and thoroughly.^12^,^16^

In previous work, we performed a systematic literature search to identify relevant QIs for the pharmacological care of older adults with CNCP.^17^ We also performed a RAND/UCLA Delphi study to ensure the face validity and feasibility of our previously identified QIs.^18^ However, to ensure the QIs’ measurement properties, clinical validation is needed.^19^

The present pre–post study aimed to determine the QIs’ applicability to the population, potential to improve medication reviews, acceptability by pharmacists, potential implementation issues and impact, thus clinically validating them. To our knowledge, this is the first attempt to establish the clinical validity of QIs for the pharmacological management of CNCP in older adults.

## Materials and Methods

### Design

The present pre–post study analysed how our QIs affected the pharmacological management of CNCP among older adult inpatients. It took place on the acute geriatrics wards of a tertiary university teaching hospital in Switzerland. The study’s first phase was conducted over 11 months, from June 2023 to April 2024.^20^ It used routinely collected data from the hospital’s clinical pharmacy service and involved weekly accompaniment on ward rounds. During this routine clinical pharmacy work, pharmacists conducted medication reviews and consulted patient histories and laboratory values, corresponding to the work of a level 2b medication review.^21^ The study’s second phase was conducted over five months, January to May 2025. In this phase, our list of QIs was applied with regard to each patient when preparing for ward rounds. We obtained informed consent from the patients in both phases prior to the start of the study. This study was exempt from full ethics approval by the Ethics Committee of the Canton Bern, Switzerland (submission numbers 2024–00596 and 2024–01252), because it does not fall under the Human Research Act, Art. 2, para. 1. All participating patients signed a general waiver consenting to the further use of their data.

### QI Characteristics

As Campbell et al proposed, we developed our QIs following a systematic search of the literature,^12^,^17^ and we sought expert consensus using a RAND/UCLA Delphi study. During this process, an expert panel evaluated the validity and feasibility of our proposed QIs. A set of 51 QIs was compiled.^18^ These covered the categories of general pharmacotherapy and the appropriate use of opioids, non-steroidal anti-inflammatory drugs (NSAIDs), paracetamol, metamizole and co-analgesics. As per Donabedian’s model, these QIs were procedural in nature. Procedural QIs measure the quality of the care delivered to patients and include, for instance, the drug treatments provided to them.^22^ We chose procedural QIs because they are the most relevant for clinical activities.^23^ Of the 51 procedural QIs initially developed, we tested 38 within the scope of this study.

QI ratios are calculated using a numerator and a denominator. The denominator represents the total population of patients to which the QI is applicable (eg those with localised CNCP). The numerator represents those in which the QI criteria are met (eg they received a topical therapy). The QI ratio reveals the percentage of patients meeting the QI criteria relative to the population it is applied to (numerator / denominator x 100).

### Outcomes

**Figure 1:**
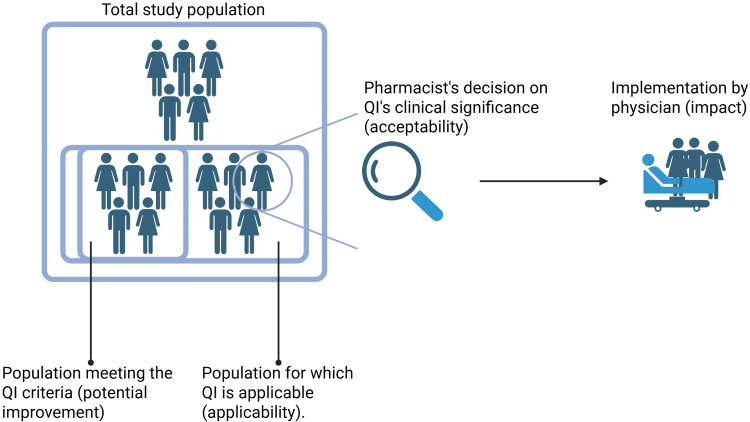
Graphical representation of the dimensions tested for the QIs’ clinical validation. Applicability: Ratio of the patients to whom the QI can be applied to the total population. Potential to improve medication reviews (QI score): Patients meeting QI’s criteria before a pharmacist’s medication review divided by the population to which it could be applied. Acceptability: Pharmacists evaluated whether the discrepancies in treatment suggestions revealed by a QI were clinically significant. Acceptability is the ratio of patients with clinically significant discrepancies over the total number of patients with discrepancies. Impact: Suggestions to mitigate unmet QIs are proposed to physicians, who then decide (or not) to implement necessary changes. The impact of each QI was calculated by comparing its score for phases 1 and 2. Created with BioRender.com.

To measure our QIs’ clinical validity, we assessed the following dimensions (see Figure 1), adapted from Fujita et al:^19^

- Applicability: We considered a QI to be “unapplicable” if the number of patients it could be applied to (the denominator) was < 5% of the participants over both study phases.^24^,^25^
- Potential to improve medication reviews: We considered a QI to have a ‘low potential to improve medication reviews’ if its criteria had already been met in ≥ 90% of participants prior to the pharmacist’s medication review.^24^,^25^
- Acceptability by pharmacists: We considered a QI to be “acceptable” if pharmacists decided that discrepancies in treatment suggestions detected by the QI were clinically significant in ≥ 50% of participants affected by that QI.
- Implementation issues: We considered any problems regarding a QI and arising in clinical practice to be implementation issues, including barriers to data collection or unintended consequences (eg overtreatment or too narrow a focus on the QI criterion).
- Impact (sensitivity to change, Fujita et al^19^): To evaluate this, we compared phases 1 (standard medication review only) and 2 (QI-enhanced mediation review) at the end of the intervention—that is, after the pharmacists had made their suggestions to physicians and the latter had decided (or not) to implement them. Specifically, we divided the number of patients who met the QI criteria by the population the QI was applicable to. If the QI criteria were met by more people in phase 2 than in phase 1 (ie the ratio of the QI’s application in phase 2 over its application in phase 1 was > 1) we considered the QI to be “impactful”.

### Statistical Analyses

To characterise the study populations in each phase, we calculated their medians and interquartile ranges (IQR) or percentages, where appropriate. We also calculated standardised mean differences (SMDs) to compare the study populations in phases 1 and 2.

## Results

In phase 1, 89 patients met our inclusion criteria, and in phase 2, 48 patients did so. The median patient age was 84 years (IQR: 78–89) in phase 1 and 83 years (IQR: 80–90) in phase 2. Women comprised 55% (n = 49) of patients in phase 1 and 58% (n = 28) in phase 2. Patients were taking a median of 11 (IQR: 8–15) drugs regularly in phase 1 and 11 (IQR: 8–13) in phase 2. The overall SMDs calculated were below 0.5, with the exception of the number of *pro re nata* prescriptions (phase 1: 7 [IQR: 5–8]; phase 2: 8 [IQR: 7–9]) and metamizole prescriptions (phase 1: 64%; phase 2: 88%). More details are shown in Table 1.

**Table 1:** Description of the Study Populations. Phase 1 Describes the Time Before the Implementation of the Quality Indicators

	Overall	Phase 1	Phase 2	SMD
Patients	137	89	48	
Age, median [IQR]	84 [78, 89]	84 [78, 89]	83 [80, 90]	0.177
Sex = female (%)	77 (56)	49 (55)	28 (58)	0.066
Residence = At home (%)	124 (91)	78 (88)	46 (96)	0.301
Drugs, median [IQR]	11 [8, 14]	11 [8, 15]	11 [8, 13]	0.213
Drugs PRN, median [IQR]	7 [5, 9]	7 [5, 8]	8 [7, 9]	0.549
Paracetamol (%)	128 (93)	83 (93)	45 (94)	0.020
NSAIDs (%)	5 (4)	3 (3)	2 (4)	0.042
Topical NSAIDs (%)	18 (13)	10 (11)	8 (17)	0.157
Metamizole (%)	99 (72)	57 (64)	42 (88)	0.569
Opioids (%)	72 (53)	48 (54)	24 (50)	0.079
SNRIs (%)	4 (3)	2 (2)	2 (4)	0.109
TCAs (%)	1 (1)	0 (0)	1 (2)	0.206
Diagnoses, median [IQR]	18 [15, 21]	17 [15, 21]	20 [17, 23]	0.437
Back pain (%)	76 (56)	45 (51)	31 (65)	0.287
Arthritis (%)	60 (44)	38 (43)	22 (46)	0.063
Rheumatoid arthritis (%)	30 (22)	24 (27)	6 (13)	0.370
Headache (%)	4 (3)	2 (2)	2 (4)	0.109
Neuropathic pain (%)	18 (13)	7 (8)	11 (23)	0.426

**Abbreviations**: IQR, interquartile range; NSAIDs, non-steroidal anti-inflammatory drugs; PRN, pro re nata; SNRIs, serotonin noradrenaline reuptake inhibitors; SMD, standardised mean difference; TCAs, tricyclic antidepressants.

Combined over both phases, 29 QIs (76%) were not met in at least one patient. Patients had a median of 4 treatment discrepancies (IQR: 3–5) and, of those, pharmacists considered a median of 1 treatment discrepancy per patient to be relevant (IQR: 0–1).

See Table 2 for an overall evaluation of the QIs and Table 3 for the QIs meeting the predefined criteria for every dimension studied.

**Table 2:** Overview of All the Quality Indicators Tested and Their Performance in the Different Dimensions

No.	Quality Indicator	Applicability	Potential to Improve Medication Reveiws	Acceptability by Pharmacists	Implementation Issues	Sensitivity to Change
G1	IF an older adult has CNCP, THEN define care goals with the patient that focus on quality of life and functionality.	100%	96%	0%		
G3	IF an older adult has CNCP, THEN monitor for adverse drug events.	100%	79%	50%		#
G5	IF an older adult has CNCP, THEN pharmacological treatment should be provided.	100%	100%			
G6	IF an older adult is being treated for CNCP, THEN choose oral drugs.	100%	98%	50%		1%
G8	IF an older adult has localised CNCP, THEN use topical drugs.	88%	29%	73%		64%
G9	IF an older adult has CNCP, THEN use sustained-release forms around the clock.	100%	21%	0%		
G10	IF an older adult is diagnosed with CNCP, THEN use base medication combined with as-needed medication.	100%	63%	2%		1%
O5	IF an older adult with CNCP is treated using opioids, THEN their effects should be monitored.	52%	100%		Y	
O6	IF an older adult with CNCP is treated using opioids, THEN they should be offered a bowel regimen or medical records should document the potential for constipation or explain why bowel treatment is not needed.	52%	86%	75%		8%
O7	IF an older adult with CNCP requires opioids, THEN pethidine/meperidine should not be used.	52%	100%			
O9	IF an older adult with CNCP is treated using opioids, THEN use long-acting formulations.	52%	39%	0%		
O10	IF an older adult with CNCP is treated using opioids, THEN use short-acting opioids for breakthrough pain.	52%	96%	0%		
O11	IF an older adult with CNCP is treated using opioids, THEN they should have tried all other options without success.	52%	100%		Y	
O12	IF an older adult with CNCP and renal impairment is treated using opioids, THEN consider using buprenorphine, hydromorphone or oxycodone.	25%	93%	33%		0%
O13	IF an older adult with CNCP requires opioids, THEN avoid codeine.	52%	96%	0%		
O14	IF an older adult with CNCP is treated using opioids, THEN other sedative drugs should be avoided.	52%	40%	12%	Y	11%
O16	IF an older adult with CNCP and hepatic impairment is treated using opioids, THEN consider hydromorphone.	1%	50%	100%		0%
O17	IF an older adult with CNCP requires opioids, THEN do not use tramadol.	52%	98%	100%		0%
N1	IF an older adult with CNCP – has a history of peptic ulcer disease or gastrointestinal bleeding and/or – currently uses antithrombotics, anticoagulants, corticosteroids or SSRIs AND they are treated using a cyclooxygenase-nonselective NSAID, THEN they should be provided concomitant treatment with a proton pump inhibitor.	4%	71%	0%		
N2	IF an older adult with CNCP has renal impairment, THEN do not use NSAIDs.	51%	93%	100%		8%
N3	IF an older adult with CNCP has peptic ulcers or gastrointestinal bleeding, THEN do not use NSAIDs.	20%	88%	100%		0%
N6	IF an older adult with CNCP is treated using NSAIDs, THEN do not use multiple NSAIDs.	4%	86%	0%	Y	
N7	IF an older adult with CNCP has heart failure or other cardiovascular diseases, THEN do not use NSAIDs.	81%	95%	100%		15%
N8	IF an older adult with CNCP is treated using NSAIDs, THEN do not combine with corticosteroids.	4%	86%	100%		50%
N9	IF an older adult with CNCP requires NSAIDs, THEN do not use indomethacin.	4%	100%			
N10	IF an older adult with CNCP has an *H. pylori* infection, THEN do not use NSAIDs.	1%	100%			
N11	IF an older adult with CNCP requires NSAIDs, THEN do not use ketorolac.	4%	100%			
P1	IF an older adult with CNCP is treated using paracetamol, THEN do not exceed a dose of 3 g per day.	93%	83%	88%		8%
P2	IF an older adult with CNCP and liver cirrhosis is treated using paracetamol, THEN avoid daily doses above 2 g.	1%	100%			
P3	IF an older adult with CNCP and chronic alcohol consumption is treated using paracetamol, THEN avoid daily doses above 2 g.	11%	82%	100%		0%
M1	IF an older adult has CNCP and low blood granulocyte counts or other agranulocytosis-inducing drugs, THEN avoid metamizole.	4%	0%	67%	Y	3%
M2	IF an older adult with CNCP is started on metamizole, THEN educate and monitor the patient for symptoms of agranulocytosis.	70%	1%	0%	Y	
C1	IF an older adult has neuropathic CNCP, THEN consider co-analgesics.	12%	75%	25%		0%
C2	IF an older adult with CNCP has closed-angle glaucoma, benign prostate hyperplasia, urinary retention, constipation, cardiovascular diseases or severe hepatic disease, THEN avoid TCAs.	81%	99%	0%		
C3	IF an older adult has CNCP, THEN avoid TCAs.	100%	99%	0%		
C4	IF an older adult with CNCP is treated using TCAs, THEN avoid high doses (eg no more than 10–25 mg of amitriptyline).	1%	0%	0%		
C5	IF an older adult has CNCP, THEN do not use carbamazepine.	100%	100%			
C6	IF an older adult with CNCP requires an SNRI, THEN prefer Duloxetine to Venlafaxine.	4%	50%	0%		

**Notes**: ^#^Was not calculable because G3 could not be evaluated retrospectively. Missing entries: values could not be calculated, because there was no data variability (eg all patients met the QI criterion), preventing the calculation of related outcomes.

**Abbreviations**: CNCP, chronic non-cancer pain; NSAIDs, non-steroidal anti-inflammatory drugs; SNRIs, serotonin noradrenaline reuptake inhibitors; SSRIs, selective serotonin reuptake inhibitors; TCAs, tricyclic antidepressants.

**Table 3:** Overview of Clinically Validated QIs

G3	IF an older adult has CNCP, THEN monitor for adverse drug events.
G8	IF an older adult has localised CNCP, THEN use topical drugs.
O6	IF an older adult with CNCP is treated using opioids, THEN they should be offered a bowel regimen or medical records should document the potential for constipation or explain why bowel treatment is not needed.
O14	IF an older adult with CNCP is treated using opioids, THEN other sedative drugs **prescribed on a regular basis** should be avoided.
P1	IF an older adult with CNCP is treated using paracetamol, THEN do not exceed a dose of 3 g per day.
M1	IF an older adult has CNCP and ● low blood granulocyte counts, ● other agranulocytosis-inducing drugs, **such as clozapine or methotrexate,** ● **other agranulocytosis-inducing diseases, such as multiple myeloma,** ● **a previous agranulocytosis under metamizole**, THEN avoid metamizole.

**Notes**: Optimisations suggested by pharmacists are shown in bold.

**Abbreviations**: CNCP, chronic non-cancer pain; NSAIDs, non-steroidal anti-inflammatory drugs.

### Applicability

Of 38 QIs tested, 27 (71%) were applicable to 5% or more of patients. The median QI applicability score was 52% (IQR: 4–86%). The eleven QIs that did not meet our applicability criterion addressed conditions that were rare in our study population. For instance, QI O16 was only applicable to older adult inpatients treated with opioids and who had a hepatic impairment. Other QIs addressed drugs that were rarely used in our population, such as NSAIDs or TCAs.

### Potential to Improve Medication Reviews

Of the 38 QIs tested, 21 (55%) had the potential to improve medication reviews for at least 10% of patients. The median QI ratio was 91% (IQR: 65–99%). QIs with a low potential for improvement were those addressing the inpatient’s overall therapy, such as defining care goals (QI G1) or using oral drugs (QI G6). QIs addressing high-risk drugs, such as tramadol (QI O17), indomethacin (N9) or ketorolac (N11), had a low potential for improvement in our population. The QIs with the highest potential for improvement addressed the use of metamizole, such as QI M1, which focused on combining the drug with other agranulocytosis-inducing drugs and low blood granulocyte levels, and QI M2, which focused on patient education on the symptoms of agranulocytosis. Moreover, QI C4, suggesting low TCA doses (eg no more than 10–25 mg of amitriptyline daily), had a high potential for improving medication reviews.

### Acceptability

Pharmacists deemed that 12 QIs (32%) revealed clinically significant discrepancies in ≥ 50% of the participants affected by that QI. The median QI acceptability rate was 17% (IQR: 0–82%). The QIs with particularly low acceptability rates among pharmacists were those on the use of sustained-release medications (QI G9 and QI O9). Conversely, pharmacists found QI P3, on paracetamol doses for those with chronic alcohol consumption, to be highly acceptable.

### Issues with Implementation

We identified six QIs (16%) with implementation issues. First, QI O5, which investigated monitoring of the effects of opioids, had a significant issue with data acquisition. Specifically, it was practically impossible for pharmacists to extract this information from patient charts, making the QI unsuitable for implementation. A second information availability issue arose with QI O11, which measured whether all other pharmacological options had been tried before starting an opioid therapy. Third, QI O14, on avoiding delivering extra sedatives in combination with opioids, caused confusion over whether only sedatives prescribed *pro re nata* should be considered. In those circumstances, pharmacists often failed to consider the treatment discrepancy clinically significant. Fourth, QI N6, which measured patients’ use of multiple NSAIDs, also posed problems as it did not specify whether they had to be oral NSAIDs. Pharmacists did not consider cases combining topical and oral NSAIDs to be clinically significant. Fifth, QI M1, on avoiding metamizole in patients with low neutrophils or other agranulocytosis-inducing drugs, was difficult to evaluate. Problems arose due to difficulties in evaluating the risk potential of drug–drug interactions. Although very rare, many drugs have the potential to lead to agranulocytosis, but evaluating the literature for every possible drug combination imposed a disproportionate effort that often led to inconclusive results. Pharmacists preferred to check laboratory values only, but they also evaluated previous drug-induced agranulocytosis. Sixth, QI M2, on educating patients about the symptoms of agranulocytosis, was difficult to implement because when the pharmacist visited, it was usually unclear whether the patient would be prescribed metamizole at home. In addition, patients were often discharged to rehabilitation centres, making it impossible to know which drugs would be taken in primary care.

### Impact (Sensitivity to Change)

Of the 16 QIs (37%) where pharmacists made suggestions, 10 (63%) had a QI ratio greater than 1, meaning that after physicians had implemented pharmacists’ suggestions based on the QI evaluations, there were fewer treatment discrepancies than after the original standard pharmacist medication reviews. The median change was 2% (IQR: 0–9%), however in the final six QIs (Table 3), the median change was 8% (IQR: 8–11). QIs without impact included those on the choice of opioid in cases of renal (QI O12) and hepatic impairment (QI O16). Some criteria on NSAIDs also had no impact, particularly those with peptic ulcers or gastrointestinal bleeding (QI N3).

## Discussion

This study clinically validated a set of QIs whose face and content validity had been established previously. To the best of our knowledge, this is the first set of QIs to be validated for older adults with CNCP. This study therefore represents an important first step towards improving medication safety in this vulnerable population. The results revealed areas requiring improvement in the pharmacological care of older adult inpatients with CNCP. This was highlighted by the median of four treatment discrepancies identified by the QIs. This justifies greater efforts to improve medication safety for these patients. After evaluating those QIs’ applicability to the population, potential to improve medication reviews, acceptability by pharmacists, implementation issues and impact, we ended up with six QIs. Due to the low number of QIs, these are relatively easy to use in standard clinical pharmacy care. In particular, integrating the QIs into an electronic algorithm within a computerised decision support system could be beneficial.^13^ However, this may require further research. The use of these QIs may improve the detection of MRPs in this vulnerable population. Future studies should evaluate whether improving medication safety for older adult inpatients with CNCP has an effect on patient-reported outcomes.

Roughly four fifths of the QIs tested were applicable to more than 5% of our study population. The low applicability of indicators involving NSAIDs, TCAs and other potentially inadequate medications for older adult inpatients might have been due to the setting. Geriatricians may be more aware of the potential inadequacies of these drugs, hence the low frequency of prescriptions.^26^ However, even if those QIs showed low applicability at the population level, they could still be relevant at the level of an individual patient.^24^ This highlights the need for personalised medication reviews.

More than half of the QIs tested showed potential for improving patients’ therapies. This was consistent with previous studies that detected higher frequencies of MRPs among older adult inpatients with CNCP.^8^,^27^ Indeed, this further strengthens our hypothesis that interventions aimed at improving medication safety in cases of CNCP are sorely needed. Pharmacists could be ideally suited to addressing this issue as part of interprofessional teams.^28^

The QIs revealed treatment discrepancies between an ideal and the actual therapy that pharmacists found relevant in roughly one third of cases. Of note, there was a high variability of acceptance rates (IQR: 0–82%). The reasons why pharmacists gave low acceptability scores to the other two thirds of cases were closely linked to issues regarding implementation, specifically, imprecise wording and data acquisition issues related to missing documentation in patient charts. Indeed, these are among the known challenges of implementing QIs.^29^ This reinforces the value of clinically validating QIs even if their face and content validity have already been established.

When comparing standard pharmacist-delivered care to the use of enhanced medication reviews using QIs, half of the QIs involving pharmacists’ interventions were impactful. The median improvement across all QIs was low (2%), but the median improvement across the six QIs with clinical validity was higher (8%). Whether this change will result in improved patient-reported outcomes is a question for future studies. Reasons leading to an unchanged QI ratio, even after the enhanced medication review, included that pharmacists had already addressed the MRPs that figured in the QIs. In this case, the QIs would still be of clinically significant. Including them could, however, lead to professional fatigue because checking QIs would take more time than performing a traditional medication review alone.^29^

The retained six QIs addressed different levels of pharmacotherapy and will require different implementation strategies. G3 revealed that approximately one fifth of older patients were suffering from an adverse drug event, which underscores the necessity to screen for them. However, implementation will require patient interviews. G8 revealed that 71% of patients with localised CNCP did not have any topical treatment. Topical drugs are considered very safe and often recommended to try, despite their limited effi-cacy.^30^ Implementation will require training pharmacists to identify localised CNCP diagnoses to recommend topical treatments on a routine basis. O6 revealed that 14% of patients on opioids did not have a bowel regimen to prevent opioid-induced constipation, a frequent adverse event.^31^ Implementation will focus on increasing sensibility of pharmacists to address this MRP. O14 found that co-prescribing of sedative medications with opioids is common, which is consistent to previous work.^8^ As many of these drugs were only prescribed PRN, pharmacists suggested to limit the QI to drugs prescribed in a regular manner. Increased attention to the sedative burden will be needed to implement this QI. P1 revealed that 17% of patients on paracetamol received doses higher than 3 g per day, which could lead to more hepatotoxicity.^32^ Implementation strategies will have to include educational measures that particularly focus on PRN and regular paracetamol doses not exceeding 3 g per day. Pharmacists suggested to adapt M1 to be easier to use and to generate more acceptable results. Even though agranulocytosis is very rare, it may have severe consequences, which justifies additional safety measures.^33^

## Limitations

The present study had some limitations. First, we tested our QIs on a relatively small number of patients in the geriatrics wards of just one hospital. The number of pharmacists evaluating the QIs was also low, and subjectivity could have affected their evaluations. These factors limit the study’s generalisability. Nevertheless, our QIs were developed using the rigorous methodology proposed by Campbell et al^12^ which included a systematic literature search and a consensus-building Delphi study. Second, the evaluation of the QI criteria’s impact used a pre–post design involving the collection of data during two different phases. This could have influenced the results. However, SMDs indicated small differences for 16 out of the 18 variables. Two variables showed SMDs > 0.5, indicating meaningful differences.^34^ Particularly, increasing metamizole prescriptions (64% of patients in phase 1, 88% of patients in phase 2) might have had an effect on the impact assessment of metamizole-related QIs. Third, although we used previously tested criteria to clinically validate our QIs,^24^ there are no universally agreed definitions for the five dimensions we tested, and, therefore, the cut-off points chosen were somewhat arbitrary. Using different cut-off points could have resulted in the selection of different QIs. Fourth, because the pharmacists knew that this was an ongoing study, a social desirability bias could have further influenced our results, particularly regarding implementation problems. Fifth, QIs must ultimately measure treatment improvements that are relevant to patients, and further research will have to investigate this.

## Conclusion

The present study investigated 38 quality indicators (QIs) for the pharmacological management of chronic non-cancer pain (CNCP) in older adult inpatients. Only six QIs (16%) met all of our predefined criteria regarding their applicability, potential to improve medication reviews, acceptability by pharmacists, implementation issues and impact. This highlights the complexity of developing QIs in this field. Applying these six QIs may improve the detection of medication-related problems through pharmacist-conducted medication reviews and may thus increase medication safety for this vulnerable population. Future studies should evaluate whether these QIs really translate into improvements in patient-reported outcomes.
